# Effects of inorganic nitrogen and litters of Masson Pine on soil organic carbon decomposition

**DOI:** 10.1371/journal.pone.0222973

**Published:** 2019-09-26

**Authors:** Xin Yu, Lin Chao, Weidong Zhang, Longchi Chen, Qingpeng Yang, Guangjie Zhang, Silong Wang

**Affiliations:** 1 Key Laboratory of Forest Ecology and Management, Institute of Applied Ecology, Chinese Academy of Sciences, Shenyang, China; 2 University of Chinese Academy of Sciences, Beijing, China; 3 Huitong Experimental Station of Forest Ecology, Chinese Academy of Sciences, Huitong, China; Sichuan Agricultural University, CHINA

## Abstract

Soil organic matter (SOM) mineralization represents one of the largest fluxes in the global carbon cycle. Numerous studies have shown that soil organic carbon decomposition was largely changed owing to the addition of litter, however very few studies have focused on the role of plant organs in the priming effects (PEs). Here, we studied the effects of different *Pinus massoniana* organs (fresh leaf, leaf litter, twigs, absorptive fine roots, and transport fine roots) on C_4_ soil respiration by applying the ^13^C isotopic natural abundance method. Results showed that the effects of plant organs on PEs were significantly different at the end of 210 days incubation, which can be ascribed to contrasting organs traits especially non-structural carbohydrates and water-soluble compounds. Transport fine roots and fresh leaf induced positive PE, whereas absorptive fine roots induced negative PE. Leaf litter did not change the native SOC decomposition. Plant organ addition can change the microbial community and result in the reduction of bacteria-to-fungi ratio. Our results suggest that with regard to determining the PE of the entire ecosystem, using fresh leaf to represent leaf litter and aboveground to represent underground is implausible.

## Introduction

Soil organic matter (SOM) mineralization represents one of the largest fluxes in the global carbon (C) cycle [1]. Forest SOMs are the biggest C pool in the terrestrial ecosystem; hence, a litter change in these SOMs will vastly affect the global C balance [2]. Understanding the factors that regulate SOM turnover is essential to predict the terrestrial feedback on climate change [3]. On one hand, fresh organic matter can form new soil organic carbon (SOC) during litter decomposition [4]. On the other hand, fresh organic substrates can stimulate the decomposition of stabilized SOC through a phenomenon called the priming effect (PE) [5].

Plant litter typically comprises different organs, such as leaf litter, twigs, fine roots, and others (e.g., reproductive organs, bark, and detritus). Fine roots occupy nearly 48% of the annual plant litter input, whereas leaf litter and twigs account for 41% and 11%, respectively [6]. The traditional definition of fine roots are roots with diameters ≤ 2 mm. This definition covered the truth that many traits vary among root orders, and differences in these traits may influence root decomposition rates. Researchers have divided the fine roots into two distinct classes, namely, absorptive (first- to third-order roots) and transport fine roots (higher-order roots) [7], to enable comparisons among functionally similar roots. However, ecological theories generally presume the plant as a whole individual and use leaf traits to represent the whole tree traits[8, 9]. This assumption overlooked the role of other organ traits in regulating the ecosystem’s C cycle to some degree. Previous studies have explored the correlation between the decomposition rates of leaf litter and other organs, but no general conclusion has been addressed yet. Freschet et al. [10] have demonstrated that for structure-related traits, such as lignin, C controls the decomposability for all plant organs. However, Sun et al. [11] studied the decomposition dynamics of the leaf litter and fine root of 35 tree species in a temperate forest ecosystem and discovered that the decomposition mechanisms of fine roots and leaf litter were different. Phenolic substances are the major controlling factors in the decomposition of fine roots, whereas stoichiometry (e.g. C:N, lignin:N) determines leaf litter decomposition. These contrasting results proved that whole-plant ecological strategies are necessary to account for the impact of different plant organs on decomposition processes.

Priming effect is characterized by the effect of adding of exogenous substrates on SOM mineralization [12]. Numerous studies have shown that soil organic carbon decomposition was largely changed owing to the addition of external organic matter, and PE has become a universal phenomenon. However, the directions of the PE induced by litter vary, showing positive [13], negative [14, 15], or no PE [16]. The added high-quality litters, such as fresh leaf and absorptive fine roots (lower C:N ratio), act as an energy source for microorganisms in degrading SOM by producing extracellular enzymes. However, according to nitrogen (N) mining theory, added recalcitrant C substrates, such as twigs and transport fine roots (higher C:N ratio), also stimulate SOM-decomposing microorganisms to chase N for alleviated nutrient limitation [17]. Furthermore, previous studies have demonstrated that the quality and quantity of litter can determine the microbial community [18]. For example, the relative abundance of actinomycetes and fungi increases as recalcitrant substrates are added [19], whereas the relative abundance of bacterial increases owing to the addition of labile substrates [20]. Bacteria that thrive on soluble C compounds can simultaneously facilitate SOM decomposition and stimulate the activity of slow-growing fungi to elevate enzyme production [21]. Yao et al. [22] suggested that the change in microbial community structure might alter the magnitude and direction of SOC mineralization. In this experiment, plant organs have distinct traits; thus, differences in magnitude and direction of the PE induced by plant organs can be observed. Very few studies have focused on the role of plant organs in the PE and microbial community structure [5].

Many experiments that study PE utilize ^13^C- or ^14^C- labeled fresh leaf. Most model designers use these PE data in representing the entire tree litter, including the above- and below-ground litter, to predict the effect of SOM decomposition to environmental change. However, this method may lack precision. Fine roots are another important litter component. Previous literature has demonstrated that root decomposition rates strongly differ among root orders and attribute litter traits [23]. However, the process of PE induction of different functional roots is not fully explored.

For the past two decades, the N deposition in China shows an increase of approximately 8 kg N ha^-1^ [24]. N deposition can affect the litter decomposition and PE by changing the litter quantity and quality [25, 26] and altering the biomass allocation strategy(i.e., aboveground vs. underground) [27]. Moreover N deposition can suppress microbial respiration [28], alleviate lower N availability and hence decrease PE according to “N-mining” [29]. However, different authors reported inconsistent effects of nitrogen addition on PE. Previous studies showed that N can enhance [30], decrease, [31], or not affect [14, 32] PE, but these studies failed to indicate the effect if different plant organs are considered.

Masson pine (*Pinus massoniana*) is a native tree species that accounts for 7.7% of the total forest area in China [33] and occupies an important state in timber production. Masson pine exists mostly in the form of monoculture forests. At present, masson pine pure forests exhibit a decline in soil fertility and biodiversity, which results in the degradation of SOM [34]. Therefore, studying the decomposition process of masson pine litters and the associated PE process is critical to better understand the mechanisms of fertility maintenance and carbon formation balance strategy in southern China.

This study aims to determine the influence of masson pine organs chemistry on the direction and magnitude of PE at the early and the later phase of litter decomposition. To do so, we selected fresh leaf (FL), leaf litter (LL), twigs (T), absorptive fine roots (AR), and transport fine roots (TR) and applied the ^13^C isotopic natural abundance method. We hypothesized that the direction and magnitude of the PE will vary with the chemistry of plant organs and differ among decomposition stages. In addition, we speculated that organs with high concentrations of easily degradable C compounds (e.g., water-soluble compounds [WSCs], cellulose, and non-structural carbohydrates [NSCs]) will deteriorate the PE in the early phase, as micro-organisms preference, whereas induce increases in PE in the later phase, as production of extracellular enzymes. Moreover, following the N-mining concept, we hypothesized that PE will decrease as external nitrogen is added. Furthermore, we also postulated that microbial community will change differently from the chemistry of plant organs, and these changes will affect PE to some extent.

## Materials and methods

### Ethics statement

The soil used in our experiment was permitted by the experimental station of Heilongjiang Academy of Agricultural Science. There were no other specific permissions required for these locations/activities because the experimental site was located at Huitong Natural Research Station of Forest Ecosystem. We confirmed that the field studies did not involve endangered or protected species and any protected locations.

### Plant materials and soil

The study site is located at the Huitong Natural Research Station of Forest Ecosystem (26°40′ to 27°09′ N and 109°26′ to 110°08′ E) in Hunan Province, China. The study area has a humid mid-subtropical monsoon climate. The mean annual temperature is 16.5°C, and the mean annual precipitation is 1,200 mm. The masson pine was planted in 1995 after clear-cutting of Chinese fir (*Cunninghamia lanceolata*), the stand densities of this forest is approximately 1200 trees ha^-1^, the forest average diameter at breast height (DBH) is 14.22 cm, and the average tree height is 9.2 m. The soil is reddish oxisol soil, which developed from shale and slate. We collected Fresh leaf (FL), Twig (T) and Fine roots from three trees with mean DBH in July 2017. The entire root system of the selected trees was excavated from the base of the trunk, with the soil intact for laboratory tests. The rhizosphere soil was washed away with deionized water. Then, the roots were divided into Absorptive fine roots (AR (first- to third-order roots) and Transport fine roots (TF (fourth- and fifth-order roots) [7]. Leaf litter (LL) was collected from litter traps in November 2016. All plant organs were dried at 60°C immediately after collection. Table 1 shows the chemical properties of the organs.

**Table 1:** Initial different organs (absorptive fine roots, transportation fine roots, fresh leaves, leaf litter and twigs) characteristics of *Pinus massoniana*.

Traits	Absorptive fine roots	Transportation fine roots	Fresh leaves	Leaf litter	Twigs
	AF	TF	FL	LL	T
Carbon (C, %)	36.00±0.09e	38.45±0.14d	50.63±0.01c	52.29±0.01a	51.02±0.02b
Nitrogen (N, %)	0.96±0.00b	0.64±0.00c	1.42±0.00a	0.37±0.01d	0.70±0.01c
Phosphorus (P, mg g-1)	0.55±0.01d	0.41±0.02e	0.96±0.02a	0.68±0.02b	0.60±0.00c
Sodium (Na, mg g-1)	0.18±0.01a	0.11±0.00b	0.04±0.00c	0.13±0.00b	0.04±0.00c
Magnesium (Mg, mg g^-1^)	1.18±0.03b	1.59±0.02a	0.88±0.01d	0.95±0.02c	0.69±0.00e
Potassium (K, mg g^-1^)	2.69±0.07d	3.32±0.04b	6.22±0.12a	1.20±0.01e	3.06±0.02c
Calcium (Ca, mg g^-1^)	3.02±0.09d	2.62±0.06e	4.57±0.05c	8.33±0.45a	5.16±0.03b
Manganese (Mn, mg g^-1^)	0.49±0.01c	0.48±0.01c	0.97±0.01b	2.21±0.08a	0.33±0.00d
Cadmium(Cd, ug g^-1^)	5.69±0.11a	1.38±0.03b	0.30±0.01d	0.80±0.03c	0.81±0.00c
Lignin(mg g^-1^)	87.28±3.19d	126.57±3.32c	143.44±0.84ab	137.67±1.84b	149.96±1.97a
Cellulose (mg g^-1^)	93.27±5.83c	170.63±3.97b	191.09±3.94a	45.42±2.71d	175.35±5.07b
Hemicellulose(mg g^-1^)	102.79±2.33b	88.82±3.21c	107.79±1.80b	100.91±1.83b	128.01±2.88a
Water-soluble compounds (%)	19.40±0.36d	29.25±0.20c	33.09±0.56a	18.91±0.42d	31.29±0.17b
Soluble sugar(mg g^-1^)	4.59±0.22e	33.24±0.56b	28.46±0.56c	12.68±0.17d	40.11±0.09a
Starch (mg g^-1^)	25.04±0.46c	30.56±0.46b	36.47±0.37a	10.01±0.20d	31.75±0.52b
Soluble phenolics (mg g^-1^)	3.71±0.12e	125.24±0.08a	26.28±0.32c	15.02±0.47d	46.66±0.81b
Total phenolics (mg g^-1^)	8.92±0.01e	75.51±0.56b	55.20±1.16c	34.16±0.25d	78.74±0.69a
Condensed tannins (mg g^-1^)	20.18±0.59d	99.85±0.43b	57.52±0.18c	21.03±0.41d	101.53±0.67a
C:N	37.44±0.10d	60.26±0.09c	35.72±0.12e	141.52±4.08a	73.03±0.98b
C:P	660.42±13.86d	949.01±33.22a	526.81±12.61e	764.07±20.25c	847.51±5.84b
N:P	17.64±0.41a	15.75±0.57b	14.75±0.31b	5.41±0.24d	11.61±0.11c
Lignin:N	9.08±0.36d	19.84±0.53b	10.12±0.03c	37.21±1.35a	21.46±0.50b
δ13C (‰)	-29.75±1.25a	-30.68±1.17a	-31.31±0.12a	-31.01±0.15a	-29.76±0.77a

Values are means and standard errors of 3 replicates.

Significant differences among the mean values of the organs in each row are indicated by different lower-case letters (p<0.05).

Experimental soil was collected from the plough layer (0–20 cm) of an agricultural site that has been planted with a C4 maize crop for over 20 years at the experimental station of Heilongjiang Academy of Agricultural Science (45°69'N, 126°62'E), Heilongjiang Province, Northeast China. The soil pH is 6.9, and the C and N concentrations are 17.3 g kg^-1^ and 1.5 g kg^-1^, respectively. The soil sample was filtered using a 2-mm mesh sieve and thoroughly mixed. The δ^13^C value of the C4 maize soil is -15.8‰.

### Experiment design

The experiment involves 2 N levels (with and without N) and 6 plant organ treatments (non-addition soil control, FL, LL, AR, TR, and T), with a total of 12 treatments. Plant organs samples were ball-milled into fine powder and sieved through a 0.25-mm-mesh sieve. The amount of each litter organs added to the soil was SOC 10% (1.7 g kg^-1^, considering a soil depth of 10 cm and volume weight is 1.1 g cm^-3^) corresponded to a realistic yearly input of litter C in masson pine plantations [35] and mineral N added was 100 ppm N. Each treatment had three replications. One hundred grams of pre-incubated soil (oven-dry equivalent) was placed in a 1-L mason jar, and each litter component was added and thoroughly mixed. Aqueous KNO_3_ (1 mL) and deionized water (1 mL) were added to serve as the inorganic and non-N treatment, respectively. The soil moisture was maintained at 60% of the water holding capacity by adjusting the deionized water for the entire incubation period. We punched two holes in the mason jar lids to install the bulkhead connectors (SMC, KQ2E06-00A; Singapore). Polyurethane tubes (TU-0604; SMC, USA) were used to link the bulkhead connectors to a manual valve (VHK2-06F-06F; SMC, Japan). The manual valve was kept open during incubation but was closed between the sampling periods. The jars were then placed in the incubator (SPX-500; Jiangnan, Ningbo, China), which is set at 25°C.

The evolved CO_2_ was captured on days 3, 7, 10, 15, 30, 60, 110, 160, and 210. We removed CO_2_ inside each jar before gas sampling by circulating CO_2_ free air, which was generated using an air compressor (ACO-318; Hailea, Guangdong, China) pumped through a soda lime column for 2 min. The jars were immediately sealed by closing the manual valve. The gas was collected 24 h after sealing using an injection syringe, which was also used to collect the air. The air was tested for CO_2_ production. The CO_2_ concentration and δ^13^C were analyzed using a high-precision isotopic CO_2_ cavity ringdown spectrometer (Picarro G2131-i Analyzer; Picarro, Inc., Santa Clara, CA, USA).

### Plant and soil chemical analysis

The oven-dried plant experimental sample was ground and then sieved using a 0.5-mm mesh before the analysis of its chemical properties. C and N are determined using element analyzer (Model CN, vario Macro Elementar, GmbH, Germany). The total P was calorimetrically measured using the phosphomolybdic blue color method, whereas metallic elements (K, Ca, Na, Mg, Cd, and Mn) were gauged using a novAA 350 atomic absorption spectrometer analyzer (Analytik Jena AG; Jena, Germany) and then digested in 1 mol of HCl. WSCs, cellulose, hemicelluloses, and lignin were determined according to the Van Soest extraction protocol [36] using an ANKOM Fiber Analyzer (Ankom Technology; Macedon, New York, USA). NSCs and soluble and total phenolics were calorimetrically determined using a modified phenol-sulfuric acid procedure [37] and Folin–Ciocalteu reagent (gallic acid as the standard), respectively. The soluble and total phenolics were investigated using a 0.5-g sample with 30 mL of water and methanol (50%), respectively, and then shaken for 2 h before filtering. The concentration of ester/ether-bound phenols was determined through sequential extraction according to the method of Tamura and Tharayi [11]. Condensed tannins (CT) were determined using the acid butanol method. Furthermore, the soil’s NO_3_^-^ concentration was tested by extracting 2 mol of KCl and applying flow injection analysis.

### PLFA analysis

At the end of the incubation, PLFA analysis was conducted to the soil samples using a modified Bligh–Dyer method [38]. Three grams of freeze-dried soil was extracted along with phosphate buffer-chloroform-methanol (0.8:1:2), and the phospholipids were separated from other lipids on a silicic acid column. The resultant fatty acid methyl esters were separated, quantified, and identified using an Agilent 6890 gas chromatograph equipped with a flame ionization detector and an Ultra-2 column. The fatty acid methyl ester and BAME controls were utilized to identify the peaks. Methyl nonadecanoate was used as the internal standard for quantifying the PLFAs. To identify the soil microbial community composition, the following PLFA designations were used: Gram-positive (G+) bacteria i15:0, a15:0, i16:0, i17:0, and a17:0; Gram-negative (G-) bacteria 17:0cy, 16:1u7c 16:1u9c, and 19:0cy; fungi 18:1u9c, 18:1u9t, and 18:2u9,12c; and actinomycetes 10Me16:0, 10Me17:0 and 10Me18:0. The sum of G+, G-, and non-specific bacteria (14:0, 15:0, 16:0, 17:0, 18:0) was used as the total bacterial PLFA. The soil microbial community structure was investigated using relative PLFA abundance (mol%).

### Calculation and statistical analysis

$$R_{t}=\Delta C/\Delta t\times 273/\left(273+T\right)\times 12/22.4\times V/100$$ (1)

where R_t_ is the CO_2_-C amount (mg CO_2_-C kg^−1^ dry soil), ΔC/Δt is the emitted CO_2_ amount per day (ppm), T is the incubation temperature (25°C), and V is the chamber volume (m^3^). The constants 12 and 22.4 are the molecular weight of C and the molar volume of an ideal gas at standard temperature and pressure, respectively.

We separated SOM-derived CO_2_-C (R_s_, mg CO_2_-C kg^−1^ dry soil) from plant organs-derived CO_2_-C (R_p_, mg CO_2_-C kg^−1^ dry soil) using the following mass balance equations:

$$R_{s}+R_{p}=R_{t}$$ (2)

$$R_{s}\times \delta^{13}C_{s}+R_{p}\times \delta^{13}C_{f}=R_{t}\times \delta^{13}C_{t}$$ (3)

where δ^13^C_s_ is the ^13^C abundance of SOM-derived CO_2_-C measured in control soils, δ^13^C_f_ is the ^13^C abundance of different organs, and R_t_ and δ^13^C_t_ are the total CO_2_-C emitted on the day of the measurement and its ^13^C abundance, respectively.

Soil (or plant organs) cumulative production of CO_2_—C (T_s_ (T_p_), mg CO_2_-C kg^−1^ dry soil) at early and later stages of decomposition was calculated by the following equation:

$$T_{s(p)}=\sum_{i=1}^{n}\left(R_{s(p)i}+R_{s(p)i+1})/2\times (t_{i+1}-t_{i}\right)$$ (4)

where R_s(p) i_ and R_s(p) i+1_ are soil (or plant organs) CO_2_-C amount at ith and (i + 1)th incubation time (mg CO_2_-C kg^−1^ dry soil), respectively; t_i+1_−t_i_ is the interval between the ith and (i + 1)th incubation time (day); and n is the number of incubation times.

The remaining proportion of litter C decomposition (L_d_, %) was calculated using the following equation:

$$L_{d}=(1‐T_{d}/M_{d})\%$$ (5)

where T_d_ is the cumulative CO_2_ (mg CO_2_-C kg^−1^ dry soil) efflux from litter during the incubation period and M_d_ is the amount of litter C added to soil (mg C kg^-1^).

The annual decomposition rate constant (k, year^-1^) was calculated by the following equation:

$$\ln(M_{t}/M_{d})=-kt$$ (6)

where M_d_ is the amount of litter C added to soil (mg C) and M_t_ is the remaining amount of litter C (mg C) at sampling time t in the year.

The PEs was calculated as

$$PE=(T_{s}\;amended\;soil‐T_{s}\;control\;soil)/T_{s}\;control\;soil\;\%$$ (7)

One-way ANOVA, followed by post-hoc LSD test, was employed to test the differences in the initial different organs characteristics, decomposition rate constant (k), the amount of organ-derived CO_2_-C, PEs, and microbial community among the treatments. Two-way ANOVA was employed to analyze the effects of plant organs, nitrogen addition, and interactive effects. Repeated measures ANOVA was employed to evaluate the effects of plant organs, nitrogen addition, and interactive on the mineralization rate of litter organs of *P*. *massoniana*; Relationships between plant litter traits and the amount of CO_2_-C from the organs or SOM were determined through linear fitting. Independent t-test was used to test the NO_3_^-^ concentration of soil with and without NO_3_^-^ addition. These processes were performed in SPSS 19.0. (SPSS Inc., Chicago, IL, USA).

## Results

### Litter decomposition

**Fig 1:**
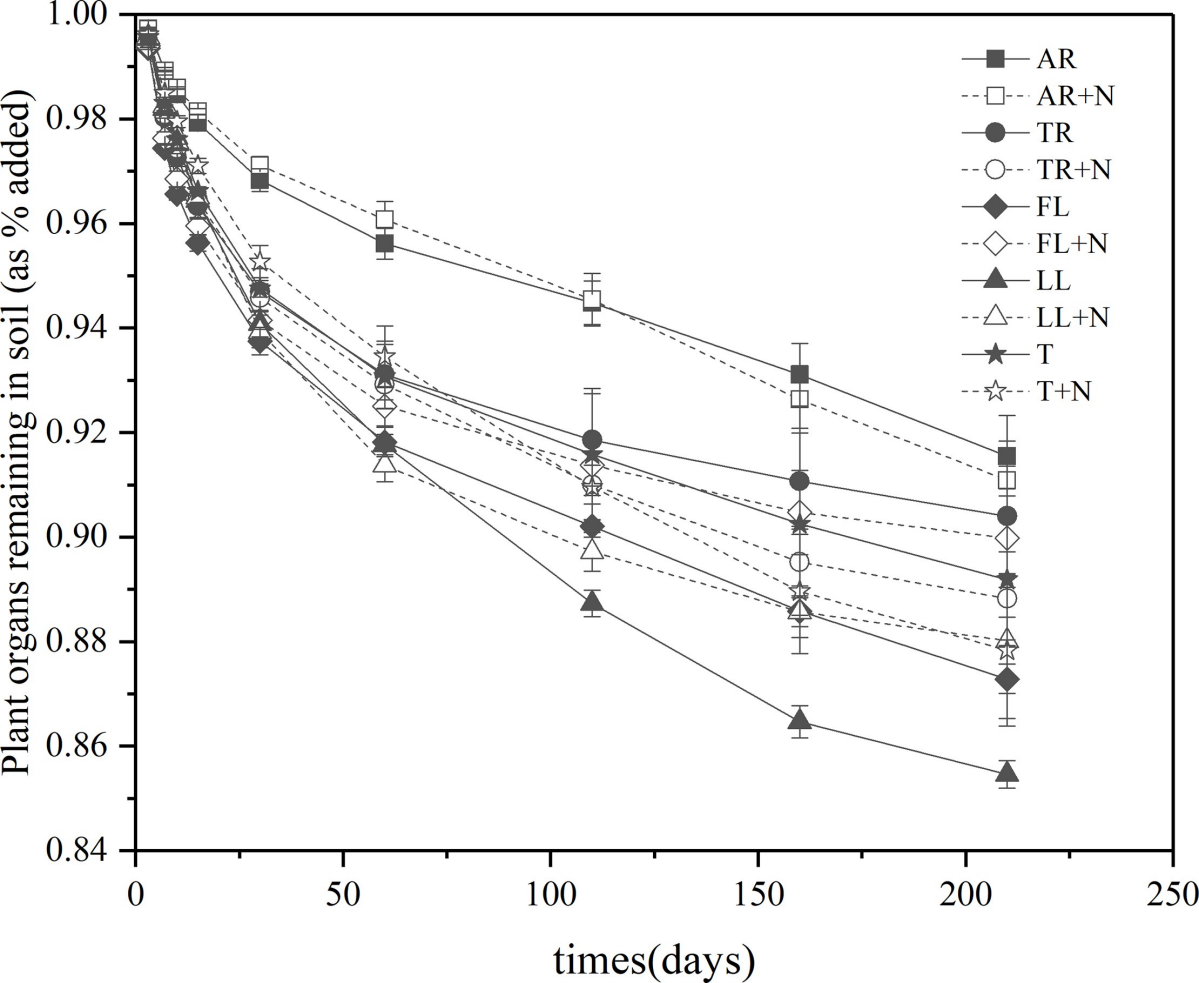
Effects of inorganic N addition on the mineralization rate of organs of *P*. *massoniana* at different incubation intervals. Vertical bars are standard errors (n = 3). Abbreviations: AR, Absorptive fine roots; TR, Transport fine roots; FL, Fresh leaf; LL, Leaf litter; and T, Twig.

**Fig 2:**
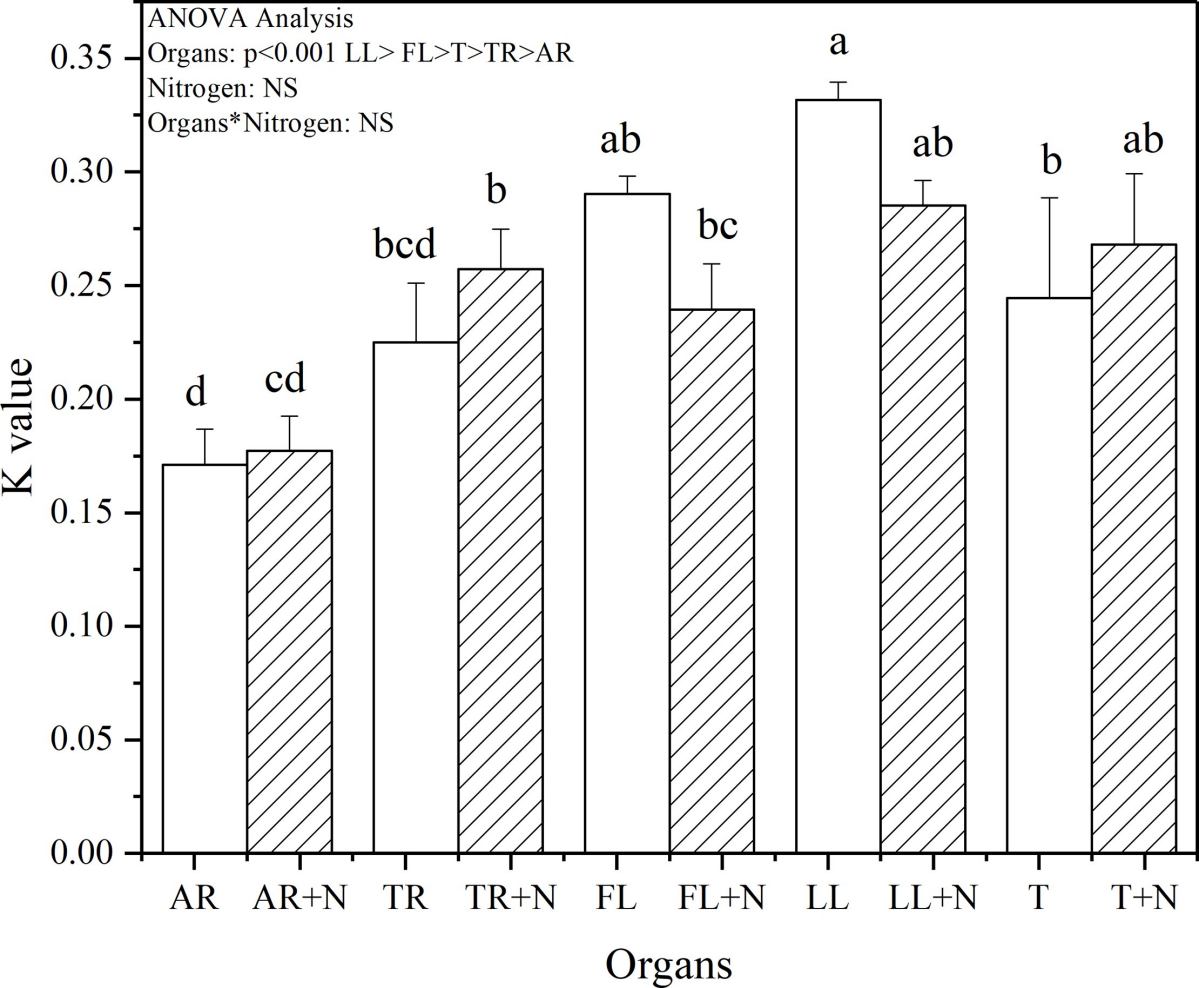
Effects of organ type and inorganic N addition on decomposition rate constant (k). Vertical bars are standard errors (n = 3). Different letters over the bars indicate statistically significant differences between treatments (P < 0.05). Two-way ANOVA result was showed above (NS: no significant difference). Abbreviations: AR, Absorptive fine roots; TR, Transport fine roots; FL, Fresh leaf; LL, Leaf litter; and T, Twig.

**Fig 3:**
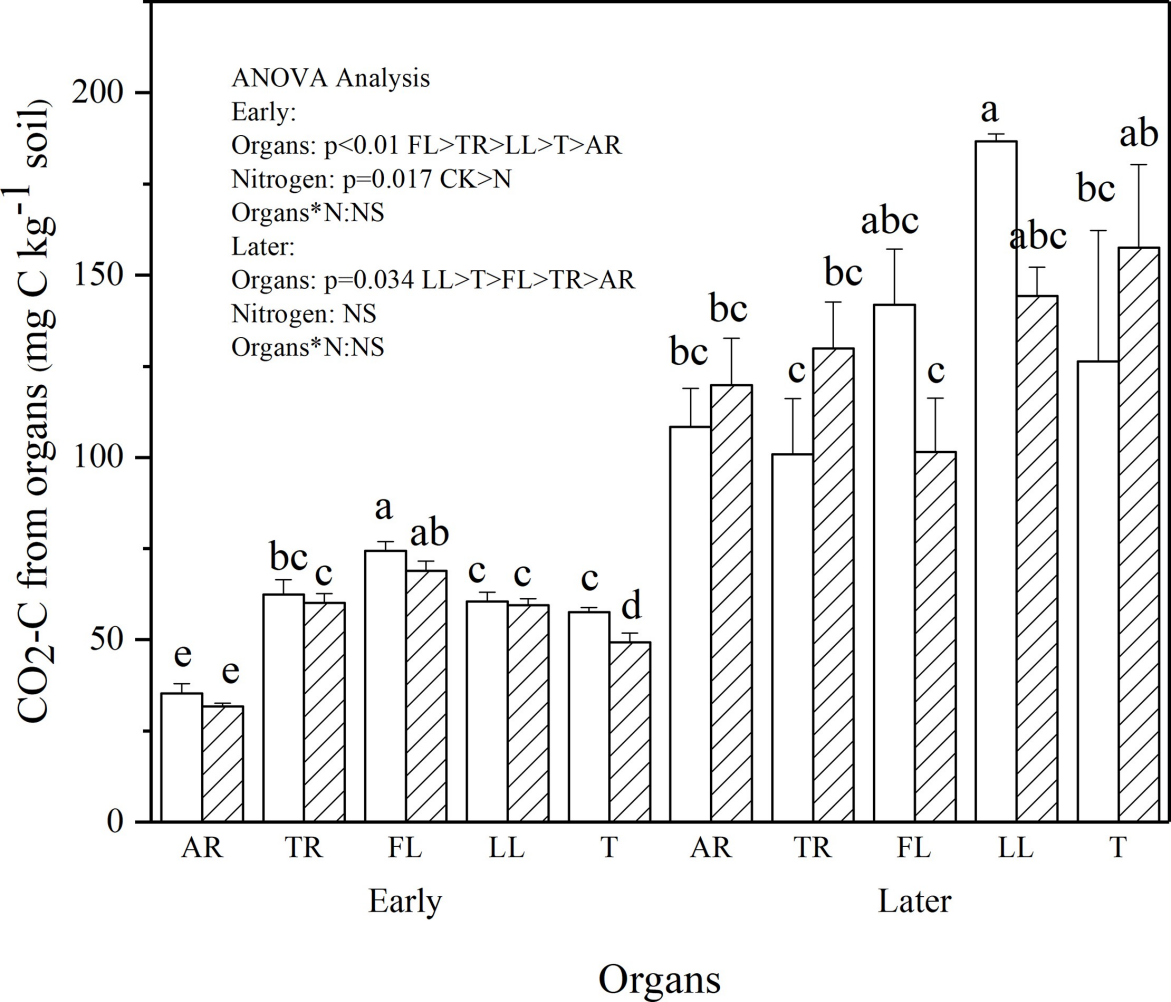
Effects of different plant organs and N addition (dash area) on the amount of CO_2_-C derived from organs at Early phase (3-15d) and Later phase (16-210d). Vertical bars are standard errors (n = 3). Different letters over the bars indicate statistically significant differences between treatments (P < 0.05). Two-way ANOVA result was showed above (NS: no significant difference). Abbreviations: AR, Absorptive fine roots; TR, Transport fine roots; FL, Fresh leaf; LL, Leaf litter; and T, Twig.

Masson pine organs all displayed a high decomposition rate in the initial stages of litter decomposition (0–15 days) and then remained low during later phase of litter decay (16–210 days) (Fig 1). At the end of the incubation, the amount of LL-derived CO_2_-C was 72.2% higher than that derived from AF. Repeated-measures ANOVA and k showed the CO_2_-C derived from plant organs was significantly affected by litter components (P < 0.001) (Fig 2 and Table 2). The addition of N did not significantly affect the organ-derived CO_2_-C (P > 0.05). In the early phase, N and litter components significantly affected the amount of organ-derived CO_2_-C (P = 0.017 and P < 0.001, respectively). The amount of FL- derived CO_2_-C was 19.2%, 22.8%, 29.3%, and 111.1% higher than that from TR, LL, T, and AR, respectively. The addition of N significantly decreased the amount of organ-derived CO_2_-C (P = 0.017), and no significant interaction between litter components and N was observed during plant organ decomposition (P > 0.05). In the later phase, only the litter components exhibited an effect on the amount of organ-derived CO_2_-C (P = 0.034) (Fig 3). In this case, the amounts of LL-derived CO_2_-C were 31.6%, 47.7%, 72.3%, and 85.1% higher than those derived from FL,T, AR, and TR, respectively.

**Table 2:** Results of repeated-measures ANOVA over time to evaluate effects of N addition on the mineralization rate of organs of *P*. *massoniana*.

Source of variation	df	*MS*	*F*	*P*
Between subjects				
Intercept	1	241.12	625244.40	<0.001
N	1	0.00	0.55	0.47
Organs	4	0.00	16.97	<0.001
N × Organs	4	0.00	0.99	0.44
Error	20	0.00		
Within subjects				
Time	1.15	0.30	827.50	<0.001
Time × N	1.15	0.00	0.14	0.75
Time × Organs	4.58	0.00	7.02	0.00
Time × N × Organs	4.58	0.00	2.17	0.10
Error	22.90	0.00		

### Priming effects

**Fig 4:**
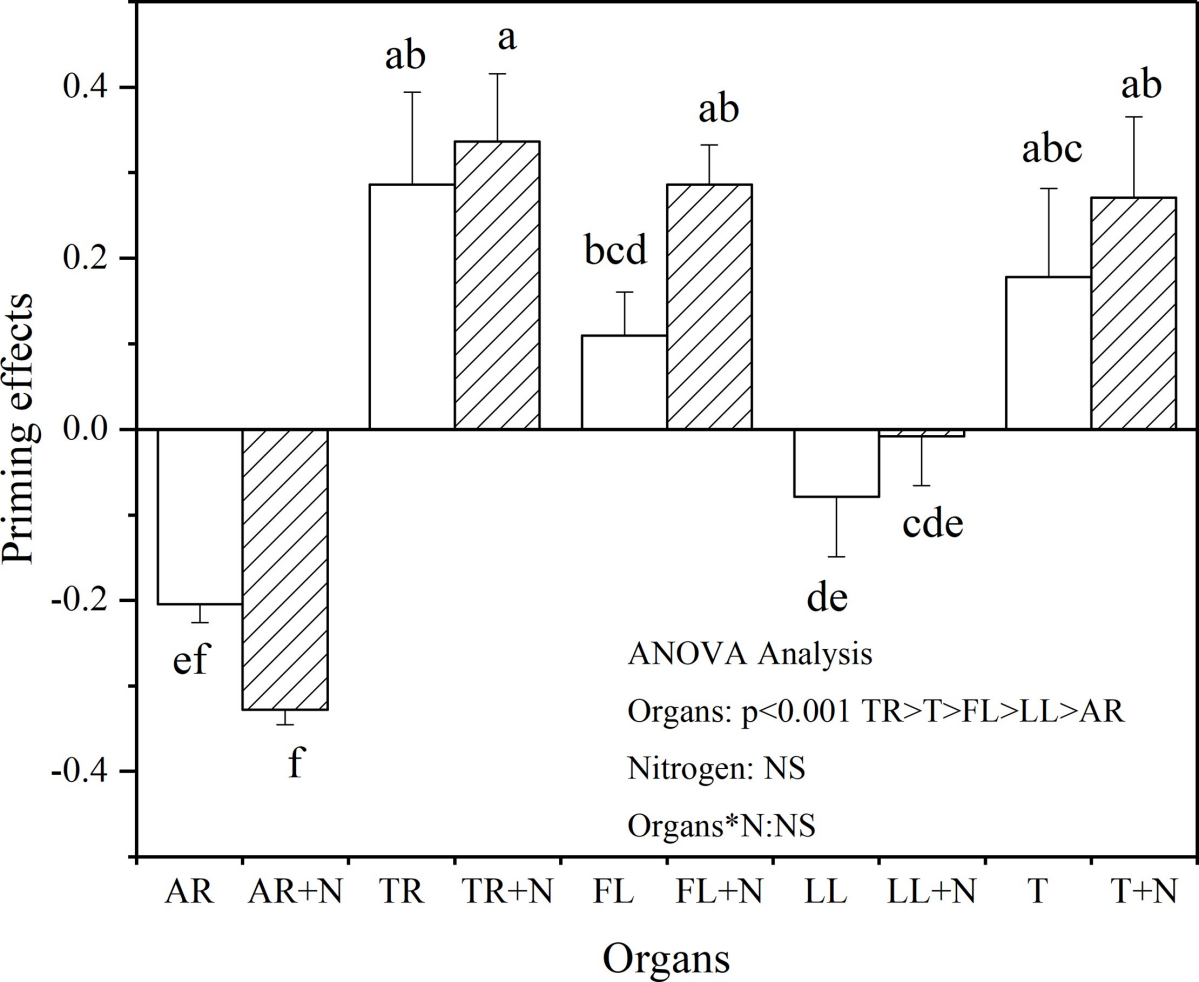
Priming effects induced by different organs and N addition (dash area) at the end of 210 d incubation. The vertical bars are standard deviation (n = 3). Different letters denote significant differences (P < 0.05). Two-way ANOVA result was showed above (NS: no significant difference). Abbreviations: AR, Absorptive fine roots; TR, Transport fine roots; FL, Fresh leaf; LL, Leaf litter; and T, Twig.

The addition of N and different litter components to the soil sample influenced the SOC decomposition to varying extents (Fig 4). To evaluate the effects of the observed PEs on the C storage of soil, we calculated the PE for each treatment using (Eq 7). The result shows that PE was significantly affected by the litter components (P < 0.001). In addition, TR and FL induced significant positive PE, whereas AR induced negative PE. We also observed that the addition of LL did not change the native SOC decomposition. Moreover, the effect of the addition of inorganic N was insignificant (P > 0.05).

**Fig 5:**
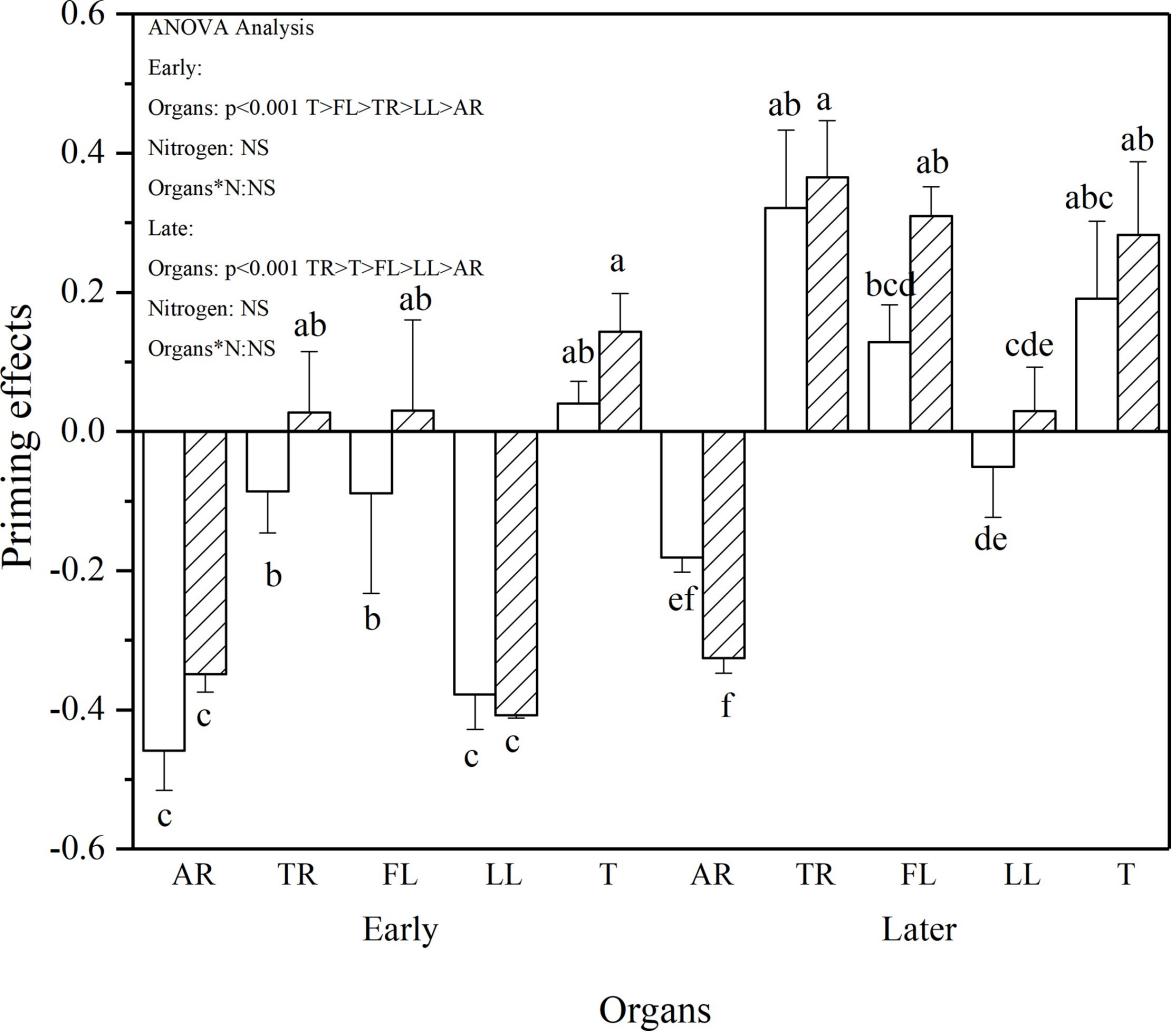
Priming effects induced by different components and N addition (dash area) at the Early phase (3-15d) and Later phase (16–210 d), respectively. The vertical bars are standard deviation (n = 3). Different letters denote significant differences (P < 0.05). Two-way ANOVA result was showed above (NS: no significant difference). Abbreviations: AR, Absorptive fine roots; TR, Transport fine roots; FL, Fresh leaf; LL, Leaf litter; and T, Twig.

The extent of PE also depends on the incubation stages (Fig 5). In the early stage of incubation, AR, TR, and LL caused a negative PE, whereas FL and T caused no PE. In the later stage, TR, FL and T caused a significantly positive PE, whereas LL and AR caused no and negative PE, respectively. In these two phases, PE was significantly affected by the litter components (P < 0.001) (Fig 5) but not by N addition (P > 0.05).

### Relationship among litter traits, decomposition, and PE

**Fig 6:**
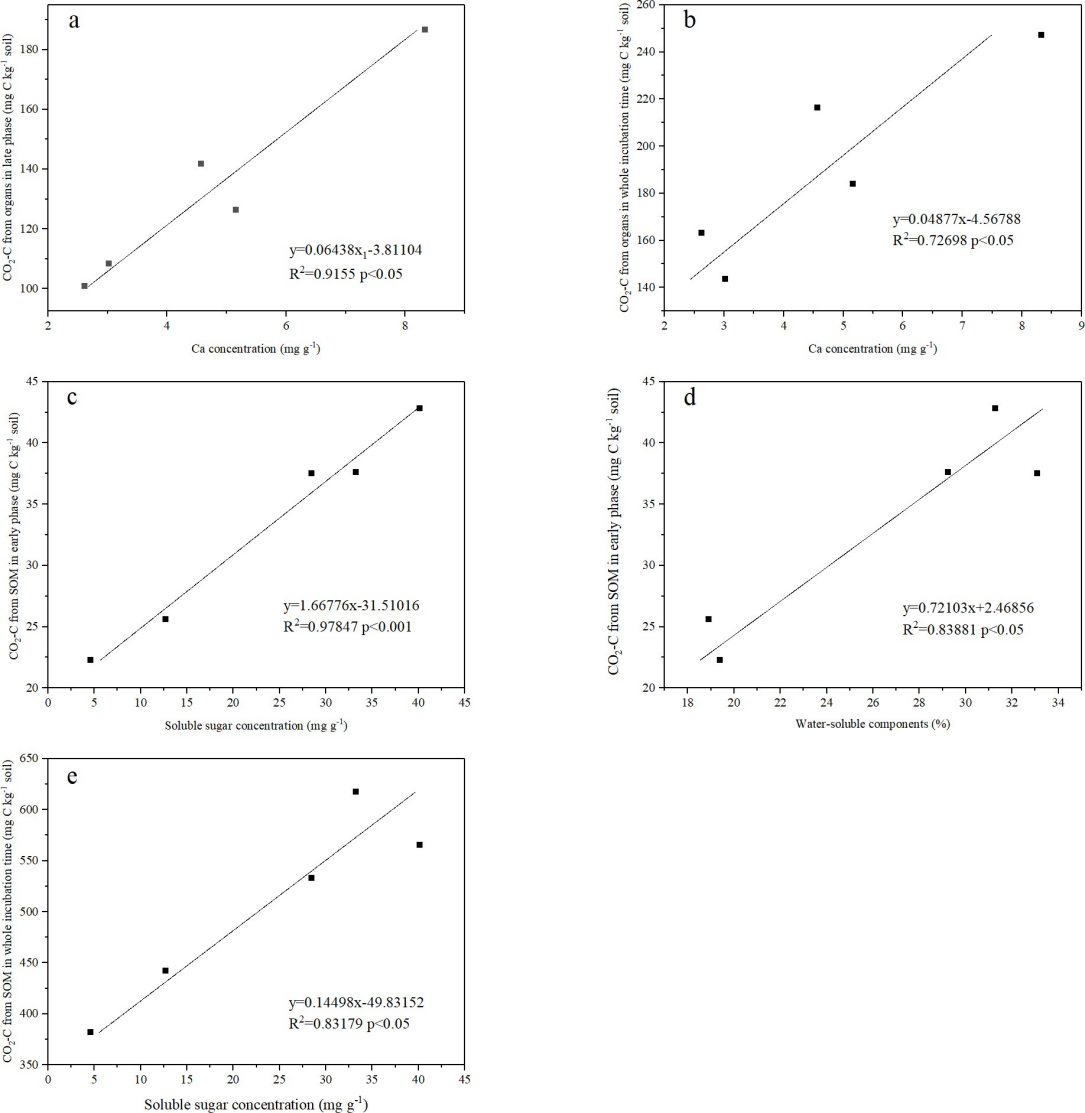
Relationships between *P*. *massoniana* litter traits and CO_2_-C from plant organs or SOM.

Different litter components have various traits. The amount of organ-derived CO_2_-C during the later phase was positively correlated with Ca concentration (P < 0.05, Fig 6A). Similarly, the amount of CO_2_-C derived from plant organs during the entire incubation time exhibited significant positive correlation with Ca (P < 0.05, Fig 6B). Soluble sugar concentration was positively correlated with SOM-derived CO_2_-C during the early phase and the entire incubation time (P < 0.05, Fig 6C and 6E), WSCs were positively correlated with SOM-derived CO_2_-C derived during the early phase (P < 0.05, Fig 6D).

### Soil microbial community

**Fig 7:**
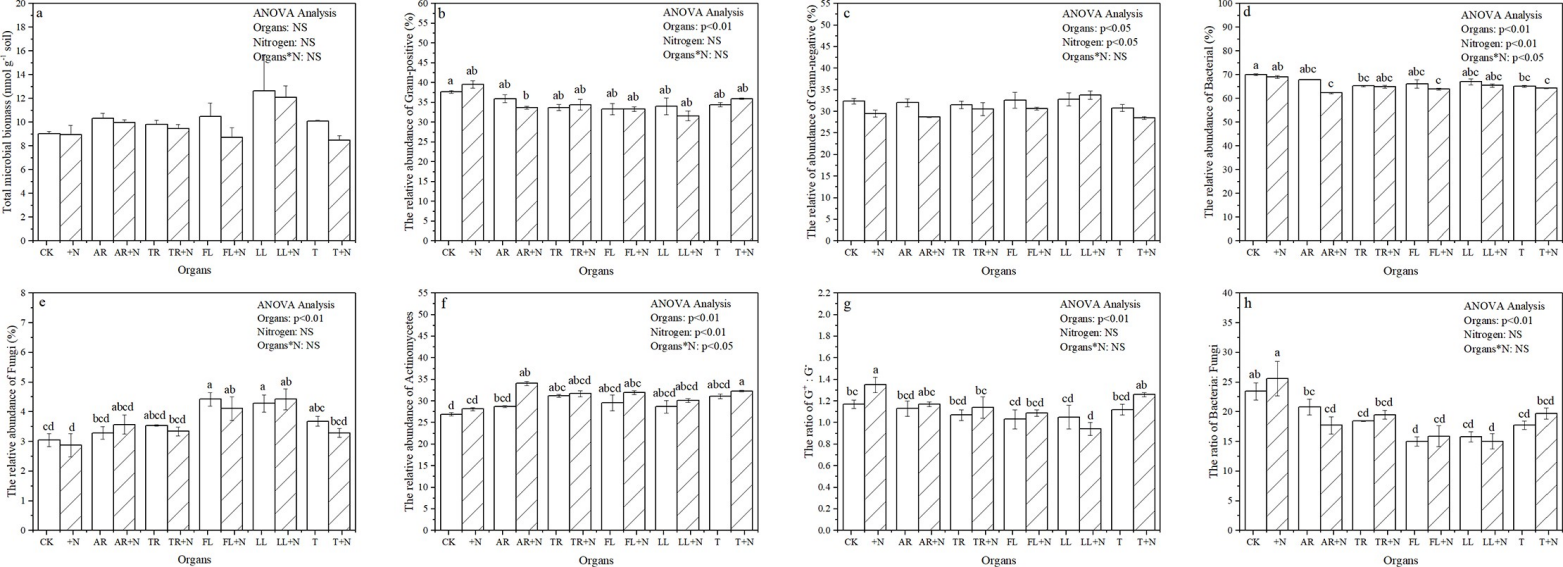
Changes in the concentration (nmol g^-1^ soil) and relative abundance (%) of PLFAs and two PLFA ratios in soils with *P*. *massoniana* organs and N addition at the end of 210 d of incubation. The vertical bars are standard deviation (n = 3). Different letters denote significant differences (P < 0.05). Two-way ANOVA result was showed above (NS: no significant difference). Abbreviations: AR, Absorptive fine roots; TR, Transport fine roots; FL, Fresh leaf; LL, Leaf litter; and T, Twig.

The addition of masson pine organs (with or without N) did not affect the total microbial biomass determined by PLFA (Fig 7A), but it significantly affected the relative abundance of G+, G-, bacteria, fungi, and actinomycetes (Fig 7B–7F). The addition of LL and FL significantly increased the relative abundance of fungi, whereas the addition of TR and T significantly decreased the relative abundance of bacteria. Consequently, the ratio of bacteria to fungi was significantly decreased (Fig 7H). Moreover, the addition of N significantly affected the relative abundance of G-, bacteria, and actinomycetes.

## Discussion

This study provides new insights on PE and the effect of dividing the litters into different plant organs on the PE-induction mechanism of the latter. Our findings demonstrate that different organs from the same individual exhibit contrasting impacts on the SOM balance owing to distinct traits. Moreover, we discovered that the PEs among FL, LL, and AR differ (positive, none, and negative PE, respectively). Therefore, the suggested traditional methods in predicting the C balance in the ecosystem are implausible.

### Effect of plant organs on litter decomposition

In this study, we found that litter component decomposition exhibits different performances during the two phases. FL decomposed faster than LL during the early phase, whereas LL decomposed faster than the other components during the later phase. This phenomenon can be attributed to the litter quality [39]. The main factors affecting the decomposition rate vary according to the phase of decomposition [40]. In the initial phase, litter mass loss is controlled by easily degradable compounds, such as WSCs and non-protected cellulose [41]. FL contains 4.2 times higher cellulose concentration and 1.7 times higher WSCs than LL (Table 1). In addition, Ca played a significant role in litter decomposition, which is consistent with the study of Berg et al. [42]. This finding was also supported by the measured Ca concentration, in which the highest amount was observed in LL. Moreover, Ca is positively correlated with organ-derived CO_2_-C (Fig 6A and 6B).

We also found that AR has slower decomposition rate than LL. This result agrees with the result of the study conducted by Sun et al. [11], in which they investigated the decomposition of first-order roots and LL from 35 woody plant species in a Chinese temperate forest. They determined that first-order roots decomposed slower than LL after six years of exposure in the field. They attributed this phenomenon to NSCs and polyphenols. In our experiment, AR contained the lowest soluble sugar and soluble phenolics (Table 1), thereby resulting in the slowest decomposition rate.

### Effect of plant organs on PEs

A significantly negative PE was observed during the early incubation stage, which is consistent with other previous works [16, 30]. This result might be because of the preferential substrate utilization of microorganisms when adding a labile C compared with SOM [43].

In the later phase, all components showed positive PE, except AR. LL and TR exhibited the highest PEs, followed by T and FL. Generally, there are two mechanisms to explain the positive PE. The first possible mechanism is “nitrogen mining” theory: if substrate lack N, microorganisms will accelerated SOM decomposition to access protected N. In our experiment, we can classify TR and T as low-quality litter (C:N = 60.2:73.03; lignin: N = 19.84:21.46, respectively). Degrading these low quality (lack N) litter will lead to a higher positive PE. However, our result showed that LL, which has the highest C:N ratio (141.52) and lignin:N ratio (37.21), have no PE during the incubation time. Therefore, the abovementioned mechanism cannot fully explain why LL exhibits no PE. The second possible mechanism is “Co-metabolism” for the degradation of other complex C compounds, microbials must assimilate labile NSCs that can provide the necessary energy for the production of various enzymes, which can co-metabolize the contemporary SOM fraction [16, 44], especially some lignin and cellulous extracellular enzymes that can degrade the soil parts that are difficult to decompose [17]. Blagodatskaya and Kuzyakov [43] also found that PEs are related to the energy input of the microorganisms in the soil. In our experiment, LL and AR contain low levels of NSCs and WSCs, thereby preventing microorganisms from achieving sufficient energy. This speculation is supported by our findings that soluble sugar and WSCs are positively correlated with SOM-derived CO_2_-C. Similar to our finding, Wu et al. [45] also declared that the fresh litter of coniferous species with high C:N ratio limits the PE.

### Effect of N on decomposition and PE

**Fig 8:**
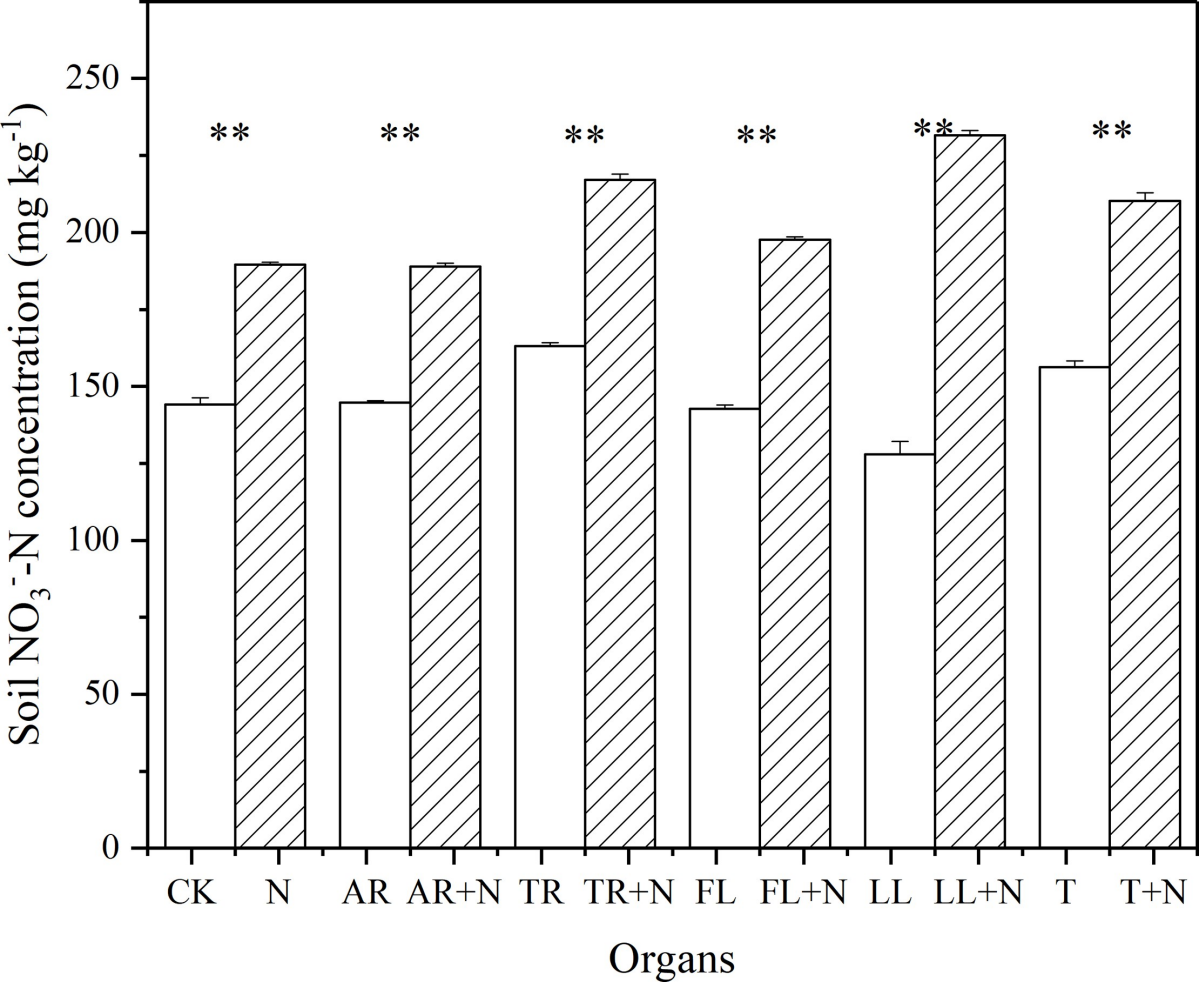
Effects of NO_3_^-^ addition on soil NO_3_^-^ concentration. Vertical bars are standard errors (n = 3). Abbreviations: CK, Control; N, Nitrogen addition; AR, Absorptive fine roots; TR, Transport fine roots; FL, Fresh leaf; LL, Leaf litter; and T, Twig. Significant differences are denoted by asterisks (P < 0.001).

In the current study, we found that adding inorganic N has a minor effect on plant litter decomposition, which might be caused by the following reason. Zhang et al. [46] declared that the effect of N on low-quality plant litter decomposition is insignificant, and *P*. *massoniana* litter can be classified as a low-quality litter. We also found that the effect of the addition of N on PE was insignificant, possibly owing to soil properties. Generally, inorganic N addition can alleviate the limitation of microbial activity, which reduces microbial N-mining and PE [31, 47]. However, our materials (C_4_ soil) have higher N concentration than normal soils (148mg/kg NO_3_^-^) (Fig 8), which can alleviate this phenomenon.

### Effects of plant organs on microbial community

Based on PLFA analyses, we determined that organ addition generally increases fungal abundance more than bacterial abundance, causing a decrease in bacteria-to-fungi ratio (Table 2) [48]. The ratio of bacteria to fungi was significantly decreased by all organ additions, except AR. These observation indicates that fungi are main actors of PE [49]. Kou et al. [50] also discovered that fungi communities will increase and use their enzyme production to decompose the SOM when the easily available substrate is exhausted. Moreover, the addition of LL and FL significantly increased the relative abundance of fungi, whereas the addition of TR and T significantly decreased the relative abundance of bacteria. This discrepancy indicates that the response of bacteria and fungi to an external C source was determined by the complexity of the C substrates [51]. Wang et al. [38] suggested that the differences in the substrate’s chemical quality can change the relative abundance of bacteria and fungi in soils, which can explain the different responses of the microbial community to the addition of different organs. Furthermore, after adding N, little or no change in microbial composition was noticed within 210 days. This result is consistent with the findings of Hicks et al. [51], where they stated that nutrient treatments are extremely small—relative to the inherent nutrient status of the soil—to change the microbial composition.

## Conclusions

This study presents important implications in the assessment of the C balance in soil by considering the relative contribution of the different organs of forest litters. The effects of plant organs on PE were significantly different, which can be ascribed to contrasting organs traits especially NSCs and water-soluble compounds and co-metabolisms (i.e., the promotion in microbial activity as a result of energy supply). TR and FL induced positive PE, whereas AR induced negative PE. LL did not change the native SOC decomposition. With regard to determining the PE of the entire ecosystem, using FL to represent LL and aboveground to represent underground is implausible. Plant organ addition can change the microbial community and result in the reduction of bacteria-to-fungi ratio. SOC decomposition by microorganisms is limited by the energy materials in nutrient-rich soil. To verify the universality of our results, we will investigate different plant varieties (coniferous and broad-leaved or evergreen and deciduous) and build an explicit model that will involve litter decomposition rates, turnover rates, and PE.
